# Apparent Death

**Published:** 1872-10

**Authors:** 


### Apparent Death.

Dr. W. H. Lathrop, Prof. of Diseases of the Brain and Mind in Detroit Medical College (*Detroit Review of Medicine and Pharmacy*), in his paper on "Apparent Death," reports one of the most remarkable and best authenticated cases of trance on record, that of William (afterwards Rev. Dr.) Tennent. While studying theology at Princeton, N. J., he had a fit of catalepsy, being in a very weak state from close application to books, without proper attention to physical exercise. After a short illness, he was regarded as dead, and the day was fixed for the funeral. His physician, however, believed him to be alive, and succeeded in having the funeral postponed from day to day for three days. The brother of Mr. Tennent then insisted that it was folly to suppose that he could be alive, and ordered that at a certain hour the burial service should occur. When the appointed time arrived, and the company were assembled, the doctor stood over his patient and asked for still another hour. At the end of that time the patient suddenly uttered a groan, and soon after gave other signs of life. His recovery was slow, and at first was attended with a loss of memory. He commenced anew the study of Latin with as much difficulty as if he had had no previous acquaintance with the language. After a considerable time his memory suddenly returned. This case is fully authenticated. The witnesses were educated persons, and all the particulars have been carefully described. Such cases, therefore, are *possible,* though extremely rare.

In conclusion, Dr. L. observes: 1st. That trance is extremely rare. 2. That apparent life in those really dead is much more common. 3d. That in doubtful cases we should wait for the appearance of decomposition before allowing the burial to take place. —*Medical News and Library.*

